# Evaluating an inclusive program for promoting equal-status participation in classrooms with high sociolinguistic diversity: diversity valuation and multilingual cooperative activities

**DOI:** 10.3389/fpsyg.2023.1257372

**Published:** 2023-12-18

**Authors:** Céline Buchs, Nicolas Margas, Marine Hascoët

**Affiliations:** ^1^University of Geneva, Geneva, Switzerland; ^2^UER AGIRS, Haute Ecole Pédagogique du Canton de Vaud, Lausanne, Switzerland; ^3^Institute of Sport Sciences, University of Lausanne, Lausanne, Switzerland; ^4^UER DEV, Haute Ecole Pédagogique du Canton de Vaud, Lausanne, Switzerland

**Keywords:** cooperative learning, equal-status participation, inclusion, sociolinguistic diversity, status among peers, primary school

## Abstract

**Introduction:**

The inclusion of students with diverse heritage languages is an emerging issue in all OECD countries due to the global rise in international migration. With regard to their large cultural and linguistic heterogeneity, primary school classes in the French-speaking region of Switzerland are extraordinary grounds to develop inclusive teaching in context of high diversity. This research-action aims to enhance students’ status among their peers and promote equal-status participation in academic activities in such classes. The research perspective focuses on valuing diversity within classes and emphasizing students’ linguistic competence through cooperative activities.

**Methods:**

The tested inclusive program places value on linguistic diversity and proposes multilingual cooperative activities that involve students’ family languages and require the contributions of all students. The research was conducted over the course of a school year, involving 3rd-4th grade students. It compared the evolution students’ status among peers (being chosen as a groupmate for play and work) from the beginning to the end of the school year in four classes with the inclusive program (*N* = 77) and four control classes without the inclusive program (*N* = 62).

**Results:**

The results indicated expected changes in status: status increased in classes with the inclusive program, while it decreased in classes without the program. Moreover, the intervention specifically supported the status of vulnerable pupils. In classes with the inclusive program, students with initially low status experienced the greatest improvement, whereas in control classes, there was no correlation between initial status and changes in status. At the beginning of the school year, across all classes, students with low status participated passively, experiencing higher levels of exclusion and displaying more discrete behavior, highlighting potential initial status-problems issues. This pattern persisted in control classes without the inclusive program, where low-status students were more likely to remain passive, while initially high- status students were more likely to become leaders. In contrast, with the inclusive program, the relationship between status and participation diminished by the end of the year.

**Discussion:**

These findings suggest that the inclusive program contributed to reducing status-related problems and promoting more equal-status participation.

## 1 Introduction

The challenge of inclusive education is to provide equitable and high-quality education for all learners (UNESCO, 2009). It not only involves ensuring access to education but also requires full participation in school life and successful educational experiences. Therefore, inclusive education can only be achieved if mainstream schools are successful in educating all children in their communities, creating welcoming environments, combating discriminatory attitudes, and overcoming barriers that hinder the participation and success of certain learners (UNESCO, 2019). This definition of inclusive education is broader than one that focuses exclusively on students identified with special needs. The classroom environment must support positive experiences for all students.

Offering quality learning opportunities for all involves both positive interactions between groups and fairness (Cañabate et al., 2021), as well as equity, which supports and embraces diversity (Ainscow, 2020). The objective is to enhance each student’s social and pedagogical participation (Forslund Frykedal and Hammar Chiriac, 2018). Inclusive education, therefore, requires teaching that addresses the needs of all students, with particular attention to those at risk of learning difficulties and dropping out. Teachers need pedagogical inclusive programs that facilitate the active participation of all students in the classroom (Farmer et al., 2019).

Cooperative learning is proposed as a means of supporting inclusion as it fosters positive relationships between students and facilitates learning for all (Juvonen et al., 2019). It creates inclusive and culturally responsive pedagogy likely to support all students and especially newly arrived students (Ferguson-Patrick, 2020), particularly relevant for classes with culturally and linguistically diverse backgrounds (Gillies et al., 2023b). However, cooperative learning is rarely implemented in classrooms (Pianta et al., 2007; Benhaïm-Grosse et al., 2020). This paradox reflects the challenges that teachers face in implementing cooperative learning, particularly in contexts characterized by high linguistic diversity, which hampers the positive interdependence necessary for effective cooperation (Cohen, 1994; Lotan, 2022). To address this issue, an inclusive program was developed in collaboration with primary teachers to accommodate the significant sociolinguistic diversity in their classes. This program includes activities that promote openness to others, openness to linguistic diversity, and multilingual cooperative activities. The latter are based on recommendations from *Complex instruction* (Cohen, 1994; Cohen et al., 1999; Lotan and Holthuis, 2021)^1^ proposed to promote equitable student learning in heterogeneous classrooms. This pedagogical approach provides multiple ability treatments explaining that multiple skills are needed to complete the task (Cohen, 1982), acknowledging and assigning competence (Cohen et al., 1988) to all students based on their contributions. In this inclusive program, teachers implemented cooperative activities in Grade 3–4 that required multiple linguistic skills and acknowledged students’ competence based on their contributions related to their heritage language. The aim of this study is to investigate the effects of this inclusive program on the changes in students’ social and academic status, and regarding the way their participation is related to their status.

### 1.1 Socio-linguistic diversity in classrooms

Each student approaches learning in a specific way. Success and failure in the classroom contribute to determining students’ academic reputation and impact their social status (Hymel and Katz, 2019). Some students possess personal characteristics that are valued to varying degrees, while others receive specialized support that can influence their social standing. Class diversity encompasses a wide range of differences among students, including individual and social characteristics.

The diversity of students in the classroom is increasingly important in today’s educational landscape. More and more students, including those with special needs and migrant students, are educated in mainstream classes (Hymel and Katz, 2019). International human migrations are continually on the rise, with the number of international migrants reaching nearly 258 million in 2017 and currently standing at 272 million, accounting for 3.5 percent of the global population (United Nations, 2019). This trend is projected to continue and potentially accelerate in the coming decades due to growing conflicts and climate change, potentially resulting in the displacement of 1.2 billion migrants by 2050 (Institute for Economics and Peace, 2020). These trends highlight the need to establish schools that can rapidly and effectively accommodate migrant students with diverse languages and cultural backgrounds. Language competence is particularly important as it influences peer acceptance, especially for children who are emergent bilingual immigrants and may face social challenges (Farmer et al., 2019). Schools have the responsibility to provide inclusive environments that accept differences for equity, especially in intercultural classrooms (Ferguson-Patrick, 2020), focusing on relationships and engagement.

While diverse school and classroom environments can enhance inclusiveness for students (Nishina et al., 2019), they can also give rise to hierarchical structures within the classrooms that undermine the inclusion process (Farmer et al., 2019). Students belong to different groups based on labels such as exceptionalities, gender, ethnicity, language, socioeconomic status, and others, which can lead to intergroup categorization. As noted by Juvonen et al. (2019), the classroom is a conducive space for the emergence of intergroup dynamics that are crucial to consider in promoting inclusion. It is therefore essential that inclusive educational practices do not result in categorization, leading to counterproductive differentiated intergroup attitudes (Iyer, 2022).

### 1.2 Cooperative learning: a theoretical consensual promise for promoting inclusion

In order to promote inclusive education, inclusive practices must be implemented in the general classroom with all students (Hymel and Katz, 2019). Cooperative learning is widely recommended for supporting inclusion (Sharan, 2010b; Killen et al., 2011; Forslund Frykedal and Hammar Chiriac, 2018; Fabes et al., 2019; Hymel and Katz, 2019; Nishina et al., 2019). Research on cooperative learning highlights its benefits for various outcomes in inclusive education, such as learning (Johnson and Johnson, 2009; Johnson et al., 2010; Kyndt et al., 2013; Johnson et al., 2014), peer relationships (Roseth et al., 2008; Choi et al., 2011; Van Ryzin and Roseth, 2018, 2019, 2022), motivation (Johnson et al., 2014), increased interest in school, and the establishment of academic learning norms (Slavin, 2015). The literature on cooperative learning offers valuable guidance on how to effectively structure group work (see Davidson, 2021 for an overview of major cooperative methods; and Gillies et al., 2023a for a current presentation of the literature) to foster students’ social and cognitive engagement (Johnson et al., 2013; Topping et al., 2017) and ensure the inclusion of all students in academic activities (Ferguson-Patrick and Jolliffe, 2018).

More precisely, effective group work requires preparing students to cooperate by explicitly developing the cooperative, social, and interpersonal skills necessary for communication and collaboration (Gillies, 2003, 2020). Another principle is to facilitate group processing (Bertucci et al., 2012; Erbil, 2020) by encouraging students to reflect on their group dynamics and ways to improve them. The teacher also needs to create a classroom climate (Wang et al., 2020) that supports promotive peer interactions. Learning the rules and social norms for behavior during groupwork supports productive functioning during group activities (Lotan, 2022). This preparation is particularly important given the competitive values promoted by society and the emphasis on school selection (Filippou et al., 2022). Students are not accustomed to cooperation, and they may be reluctant to cooperate and revert to competitive behaviors despite cooperative instructions (Buchs et al., 2021). Establishing a safe environment where students feel comfortable to cooperate and gradually learn to cooperate is essential. Interpersonal communication and helping skills promote a sense of community, while explicit discussions about cooperative values encourage acceptance of diversity (Sharan, 2017). This preparation contributes to the development of social competence and prosocial behaviors that support inclusiveness (Nishina et al., 2019) and create conditions for students to participate safely (Batelaan and van Hoof, 2006). This aligns with the “Meet-Up” strategy proposed by Fabes et al. (2019), which addresses social norms and peer interactions at the classroom level.

In addition to this preparation, cooperative learning proposes principles for structuring student interactions in small groups to promote equal participation (Johnson et al., 1998; Kagan and Kagan, 2009; Davidson and Major, 2014; Gillies, 2016; Gillies et al., 2023a). The first principle is to create positive interdependence among learners working toward a common goal, so that students perceive a positive correlation between their success (Butera and Buchs, 2019). The teacher also needs to emphasize individual accountability and responsibility, making everyone’s contributions necessary and valued (Topping et al., 2017). Finally, working in small groups facilitates each student’s participation, while the cooperative structure maximizes students’ engagement and contributions (Sharan, 2010a). This structure encourages the integration of all students’ resources and respects their contributions in order to achieve learning goals (Sharan, 2017). Equal participation is a major issue for cooperative learning in order to sustain successful experience for all students (Kagan, 2021) and need to be structured.

This cooperative pedagogy aligns with the principles of “Universal Design for Learning” in education, which advocates for whole-class activities that emphasize both academic and social participation of students (Hymel and Katz, 2019). By working together toward a common goal, students develop a sense of belonging to the same group, which can help reduce social categorization (Cohen, 1994). Creating opportunities for positive interactions between different groups is likely to decrease stereotypical perceptions and potential discrimination (for review, Pettigrew and Tropp, 2006; Hewstone and Swart, 2011), while fostering more inclusive social identities (Reimer et al., 2022).

### 1.3 Cooperative learning: a challenging practice

A first paradox arises with the low implementation of cooperative learning in classrooms (Baines et al., 2003; Abrami et al., 2004; Pianta et al., 2007; Buchs et al., 2017; Abramczyk and Jurkowski, 2020). Despite the documented benefits and the established guidelines, cooperative learning in classroom remains a challenge (Sharan, 2010a). The effective implementation of cooperative procedures is complex (Jolliffe, 2015; Ferguson-Patrick and Jolliffe, 2018), requiring significant changes in teaching practices (Gillies and Ashman, 2003). Teachers may encounter difficulties (Abrami et al., 2004; Gillies and Boyle, 2010; Jolliffe, 2015; Veldman et al., 2020) and may struggle with proper implementation (Antil et al., 1998; Sharan, 2010a), which can diminish the positive effects on students’ social acceptance (e.g., nominations by classmates as friends or groupmates, Klang et al., 2020) and learning outcomes (Hattie, 2009; Topping et al., 2017).

A second paradox emerges from the fact that, in the absence of a rigorous cooperative structure, group work has the potential to exacerbate learning gaps among students. Some students tend to be more confident and comfortable expressing their opinions, ideas, and contributions during group activities while other may feel less confident or valued within the group, leading to reduced participation. This issue refers to status among peers, the social standing holds within a group of classmates. Student status is influenced by various characteristics (Cohen, 1994; Lotan, 2022), including diffuse characteristics (e.g., gender, cultural and social backgrounds), specific characteristics (e.g., specific skills or abilities), and, most importantly, local characteristics related to academic status and popularity. Based on status, students develop academic and social hierarchies, where classmates perceive themselves and are perceived by others as more or less competent (Lotan, 2006). These expectations regarding competence influence actual participation, with some students and more likely to participate during group work based on their respective status. In highly diverse classrooms, high-status students tend to participate more and take on the role of facilitators (Cohen and Lotan, 1995). This pattern of interaction is referred to status problems, i.e., the correlation between students’ status and their participation (Cohen and Lotan, 1995; Lotan, 2022).

Because participation serves as an indicator of inclusion (Forslund Frykedal and Hammar Chiriac, 2018) and determines learning (Cohen, 1994; Mercer, 2008; Webb et al., 2021; Lotan, 2022), ensuring equal-status participation is particularly relevant in heterogeneous contexts. Without precautions taken in group work, status problem leads to unequal participation, creating a virtuous/vicious cycle that perpetuates and widens the initial hierarchy in classrooms. Cohen and Lotan (1995) and Lotan (2006) warned that unless these issues of unequal status and participation are addressed in detracked heterogeneous classrooms, inequality will persist. This is particularly important because the frequency of teachers’ use of cooperative learning is not always associated with the quality of teachers’ implementation in class (Abramczyk and Jurkowski, 2020).

Thus, one main challenge faced by teachers in implementing cooperative learning for supporting inclusion is ensuring equal status among students in the class, considering their social groups outside the classroom (Killen et al., 2011; Farmer et al., 2019) and their status among peers inside the class (Cohen et al., 2004; Lotan and Holthuis, 2021; Lotan, 2022). Equal status is needed to facilitate participation of all in academic tasks (Cohen, 1994; Pescarmona, 2015), to develop inclusive education in heterogeneous classrooms (Cohen, 1994; Lotan, 2006; Pescarmona, 2014; Lotan and Holthuis, 2021; Lotan, 2022) and to give voice to diversity (Pescarmona, 2023). It requires fostering the participation and learning of those who have lower initial status (Cohen and Lotan, 1997). This is particularly crucial and challenging for students who have not yet acquired all the social and/or academic skills or have limited mastery of the language of instruction (Cohen et al., 1999).

### 1.4 Supporting students at risk in cooperative learning

#### 1.4.1 Supporting students’ competence expectancies

In order to support the participation of all students, teachers need to reinforce students’ competence expectancies, especially for students who are at risk (Cohen, 1994; see Lotan, 2022 for a review). First, this can be achieved by highlighting the competence of specific students who have a lower status. Teachers can design activities that allow students to showcase their specific skills and abilities, providing them with opportunities to demonstrate their competence. When students are able to showcase their abilities and make meaningful contributions, and teachers publicly acknowledge their accomplishments providing specific feedback, it boosts their status among their peers. Cooperative work provides teachers with the chance to observe students’ abilities and recognize their valuable contributions. Teachers can also assign specific roles during group work that align with these abilities.

Secondly, appropriate tasks should support the participation and learning of students who may be in a vulnerable position within the group due to their status (Cohen and Lotan, 1997). Engaging in challenging learning tasks helps broaden and deepen students’ and teachers’ understanding of intelligence (Lotan, 2006; Lotan, 2022). Teachers can encourage students to work cooperatively on learning tasks that require multiple abilities, extending beyond the traditional academic skills of reading, writing, and math (Cohen, 1994; Cohen et al., 2004). These tasks demand various intellectual abilities, increasing the chances that every student can demonstrate at least one specific ability. Since no student possesses all the required abilities, cooperation becomes essential to solve the task and value the contributions of all students. This approach effectively shifts expectations of students by providing meaningful opportunities for participation. Cooperative activities that involve multiple abilities offer a platform to highlight the relevance of students’ contributions to the activity (Cohen, 1994; Lotan, 2006).

The frequency with which teachers employ these two strategies aimed at reinforcing students’ competence expectancies has been shown to decrease status problems (Cohen and Lotan, 1995). Lotan (2022) offers a comprehensive review of the impacts of complex instruction on learning outcomes. The findings emphasize the significance of social interaction in the learning process, encompassing academic domains, language of instruction, and students’ disciplinary discourse.

#### 1.4.2 Heritage language in a context of sociolinguistic diversity

To promote the value of sociolinguistic diversity, it is important to design activities that align with students’ linguistic skills. Multilingual educational approaches, which recognize language as an integral part of students’ cultural identity, are instrumental in fostering inclusion (Batelaan, 2000). In classrooms characterized by high sociolinguistic diversity, incorporating students’ heritage languages offers opportunities for their meaningful contributions (Batelaan, 2000) and fosters an appreciation for the richness of differences within the classroom (Ferguson-Patrick and Jolliffe, 2018). By encouraging students to build upon their knowledge and skills in their heritage languages, cross-cultural communication is sustained, and students develop multicultural communication competencies (Gay, 2002). It emphasizes the importance of heritage language background in the development of linguistic competence (Coste et al., 2009).

Providing instruction focused on heritage languages during the early years of schooling has a positive impact on learning outcomes (UNESCO, 2009). It helps students establish meaningful connections between the curriculum and their personal experiences, which facilitates learning (Gay, 2002). This approach also demonstrates institutional recognition of the value of heritage languages by placing them on an equal footing with the language of instruction.

### 1.5 An inclusive program

In accordance with the principles of intercultural education (Batelaan and van Hoof, 2006; Berry and Sam, 2013) and *Complex instruction* (Lotan, 2022), the inclusive program incorporates the values of diversity and promotes equality and equitable participation (Buchs and Maradan, 2021). What distinguishes this program, is its focus on showcasing students’ plurilingual skills in the classroom and proposing multilingual cooperative activities that engage their family languages, thereby representing tasks that require multiple abilities (Cohen, 1994).

The objective of the program tested in this study is to provide students with equal opportunities to contribute while considering their backgrounds, particularly their competence in heritage languages. To ensure students’ comfort and contributions, a questionnaire was sent to families to inquire about the specific language or dialect students would like to use in classroom activities, their proficiency levels in this language (for reading, speaking, and writing), and whether someone could assist with homework designed to prepare students for their contributions. Some students indicated multiple languages, while others identified languages spoken by relatives beyond their immediate family (e.g., cousins, aunts, or uncles). Therefore, the program encompasses family languages in a broad sense.

Even when teachers recognize the importance of addressing status disparities in their classes, they may feel daunted by the task (Pescarmona, 2015). Furthermore, teachers in the context of the study have reported difficulties in conceptualizing and designing cooperative activities (Buchs et al., 2017) and may not feel comfortable introducing linguistic diversity into their teaching (Akkari et al., 2011). Therefore, it was crucial to provide teachers with specific activities that they can implement and support them throughout the process. To address these challenges, a set of activities was collaboratively constructed in partnership with primary teachers before this study. These ready-to-use activities were introduced to the inclusive program tested in this study.

The research team took responsibility for documenting the family languages/dialects that students could work with in the classroom. Teachers had previously received training in structuring cooperative activities (provided one year before the study at the school level). Additionally, they received one additional day of training to participate in the research. This training focused on the program’s objectives, the significance of students’ status for their participation and learning, and the issue of status disparities and status problems. Teachers were provided with all the necessary materials, instructions, and scripts for each activity, including required translations when needed. To transition smoothly into multilingual cooperative activities, preliminary activities were proposed from September to February, followed by the implementation of cooperative multilingual activities from March to June. Based on these elements, the program was structured into three stages presented below. The inclusive program materials are available upon request by contacting the corresponding author.

#### 1.5.1 Activities for opening to others

First, several activities aimed at fostering openness toward others by strengthening classroom cohesion and promoting a cooperative climate were implemented from September. These activities were designed to facilitate the discovery and acquaintance of students, establish inclusive social norms (Lotan, 2022), and emphasize cooperative values (Fabes et al., 2019). Additionally, they aimed to develop interpersonal communication and supportive skills (Sharan, 2017), social competence, and prosocial behaviors (Nishina et al., 2019).

Teachers proposed cooperative activities related to academic subjects in order to familiarize themselves and students to cooperative learning. This initial phase was designed in accordance with the cooperative framework for preparing students to cooperating and structuring cooperative work (Topping et al., 2017). Its purpose was to help students feel accepted and comfortable when participating, while also addressing the challenges associated with a competitive classroom environment (Gundara and Sharma, 2013; Buchs et al., 2021). Moreover, this approach aimed to increase the likelihood of cooperative practices (Filippou et al., 2022).

#### 1.5.2 Activities for opening to linguistic diversity

Next, activities dedicated to promoting openness to linguistic diversity were introduced from December to February. These activities were derived from regular teaching methods employed in the French-Swiss area to foster language inclusivity in schools (Perregaux, 1998; Sanchez-Mazas et al., 2019). While these methods are available to all regular teachers, the implementation of related activities in mainstream classrooms is relatively uncommon. The inclusive program introduced some of these activities.

These activities were specifically designed to cultivate positive attitudes toward plurilingual students and enhance learning in the language of instruction. Some activities focused on linguistic diversity in a general sense, while others emphasized and celebrated the actual linguistic diversity within the targeted classrooms (Perregaux et al., 2003; Sanchez-Mazas et al., 2019). The approach to embracing other languages involved listening, observing, and comparing oral or written texts in different languages during classroom activities. This provided opportunities for students to engage with the language of instruction through other languages and develop metalinguistic skills, as well as reflection on language itself. Consequently, students were equipped with the knowledge and skills necessary to welcome new and unfamiliar languages.

Beyond linguistic aspects, these activities facilitated the development of intercultural skills by exposing students to alternative ways of expression, action, and thought, while fostering positive attitudes toward languages and their speakers (Candelier, 2003; Armand and Dagenais, 2012; Coste, 2013). The activities dedicated to embracing linguistic diversity within the classroom not only supported positive relationships among classmates (Batelaan, 2000; Ferguson-Patrick and Jolliffe, 2018) but also promoted stronger connections between families and schools, enhancing student engagement in school activities (Gay, 2002).

#### 1.5.3 Multilingual cooperative activities

From March to June, a total of 22 multilingual cooperative activities were conducted. The cooperative structure, devised by the research team, ensured that each student’s contribution was crucial in achieving the common goals of the team. Some activities utilized dual-language printed materials, with each student receiving materials in their family language and French (the language of instruction), while others involved words provided by family in their respective languages. They actively incorporated the participation of students’ and parents’ cultures in classroom activities. The nature of the activities required students to draw upon their unique resources, such as specific linguistic skills for students who spoke a language other than French, or different types of contributions for students who only spoke the language of instruction. For students who had no foreign language background at home or in their relatives (2 to 6 students in each class), various alternative contributions were introduced. This included learning braille, searching for definitions in French, or assuming different necessary responsibilities, ensuring that every student’s contribution was essential during multilingual cooperative activities. Students switched teams for each activity, fostering diverse interactions and contact with different languages.

Each class consisted of 19 to 22 students. The linguistic diversity in these classes was substantial, ranging from 10 to 14 additional languages alongside French when taking into account students who spoke French at home with their parents but had a foreign language background (3 to 7 students in each class). In total, there were 27 different languages represented, including Albanian, German, Amharic, English, Arabic, Chinese, Sinhalese, Haitian Creole, Dari, Spanish, Italian, Japanese, Kinyarwanda, Konkani, Kurdish, Luganda, Norwegian, Portuguese, Romanian, Russian, Slovak, Somali, Swiss-German, Czech, Thai, Tigrigna, and Turkish. In some cases, parents did not speak French at all (1 to 6 students in each class), requiring the translation of parental authorizations. For students who spoke two different foreign languages (3 to 6 students in each class), they were given the choice of which language to use in school activities. Around 8 to 10 different languages were utilized in each class for the activities. The research team managed the relationship with translators to provide all the necessary materials for the activities, along with the French version for the teachers to identify different passages.

These activities, encompassing multiple abilities in line with complex instruction (Cohen, 1994; Lotan, 2006; Lotan and Holthuis, 2021; Lotan, 2022), allowed every student to make unique contributions toward the common goals. Teachers ensured that each student fulfilled their role and contributed to the team’s success, fostering positive team experiences. The activities were designed to value all students’ skills and publicly recognize the competence of each student. The program drew upon several practices and instructional strategies recognized as valuable and effective in culturally diverse contexts (Allison and Rehm, 2007) and addressed the characteristics of status-problem treatment (Cohen, 1994; Lotan, 2022). Importantly, while the multilingual cooperative activities provided an opportunity to value students’ skills in their family languages, they also celebrated other skills, allowing each student to showcase their abilities. The inclusive program targeted all students, with special attention given to students at risk without explicitly identifying or naming specific individuals or groups to avoid stigmatization or categorization.

The overarching hypothesis is that this inclusive three-stage program will (1) enhance students’ status among their peers, particularly for those who initially had low status, and (2) contribute to more equitable and equal-status participation in classroom activities.

The first series of hypotheses pertained to the effect of the inclusive program on the evolution of status.

*H1a*: It was expected that the inclusive program would enhance the status of students, with greater improvements observed in classes that implemented the program compared to classes without the program.

*H1b*: Additionally, it was hypothesized that the inclusive program would have a particularly positive impact on students who initially had low status. This hypothesis suggests a stronger negative relationship between initial status and the evolution of status with the inclusive program.

The second series of hypotheses aimed to investigate the role of the inclusive program in the evolution of status problems. Status problems were examined through the relationship between students’ status and their type of participation during the unstructured activity. Status problems could be identified through: (a) positive relationships between status and assertive types of participation (e.g., high-status students being more likely to endorse leadership and engage in co-construction), and (b) negative relationships between students’ status and passive types of participation (e.g., low-status students being more likely to be discrete and excluded). This approach to status problems implies the following hypotheses:

*H2a*: At the outset of the academic year (pre-test), students in highly diverse classrooms may exhibit a pattern of status problems. To investigate this hypothesis, the relationship between students’ initial status (pre-test) and their type of participation at the beginning of the year (pre-test) was examined.

*H2b*: By the end of the year, it is hypothesized that if status problems were present during the pre-test, they may persist in classrooms without the inclusive program. However, in classrooms with the inclusive program, it is expected that such problems would be diminished. Consequently, participation should not be associated with any specific status with the inclusive program. To test this hypothesis, the relationship between students’ final status (post-test) and their type of participation at the end of the year (post-test) was examined in the two conditions.

*H2c*: At the end of the year, the inclusive program is expected to disrupt the connection between initial status (pre-test) and participation at the end of the year (post-test). In other words, the inclusive program should facilitate equal-status participation across different status levels, and initial status should no longer be linked to distinct types of participation with the inclusive program.

## 2 Methods

### 2.1 Participants

The study was conducted during the 2018–2019 academic year in Geneva Canton, French-speaking area from Switzerland. In 2019, 45% of pupils enrolled in compulsory education in Geneva spoke a first language different from the language of instruction, and 44% belonged to a different nationality.^2^ The proportion of parents who held senior managerial and executive positions varied from 22.8% for French-speaking children to 15.4% for children speaking another language at home. Similarly, the proportions ranged from 53.9 to 34.0% for self-employed individuals, employees, and middle managers, and from 23.3 to 50.5% for workers or those with no occupation indicated.^3^ In terms of academic performance, the success rate at the end of the 4th grade’s cantonal exams in French was 87.3% for French-speaking pupils and 77.3% for students who spoke another language at home during the 2018–2019 academic year.

In terms of school structure in Geneva, when children arrived from foreign schools without proficiency in French, they spent half of their time in a specialized class dedicated to learning French and the other half in a mainstream class with peers of the same age. This arrangement typically lasted for one to two years. All students involved in the study were regular students in the classes included in the research. All students with parental authorization from the 8 classes were included in the study.

The inclusive program was implemented by four regular teachers in their mainstream class from one school (referred to as School A), with a total of 77 students whose parents provided authorization and participated in the pre- and post-tests. Four control classes were also included in the study, with one in School A and three in School B, comprising a total of 62 students with parental authorization present during the pre- and post-tests. Both schools were located in the same area, with 37.4% of pupils coming from modest backgrounds and 57% of students speaking a language other than French (the language of instruction) in 2019. Specifically, in School A, 49% of pupils spoke another language at home in 2016, and 39% were from modest backgrounds (compared to 55 and 48%, respectively, for School B). All participating teachers had prior training in cooperative learning and volunteered to participate in the study.

This paper focus on equal-status participation in classroom. In the present study, students’ participation was coded based on video-recorded interactions during a non-structured activity conducted in triads at the beginning and end of the school year. Only students with parental authorization for video recording were videotaped and included in the analysis. The triads consisted of heterogeneous groups with one student of low, one of medium, and one of high initial status. If a student was absent, the remaining dyads were excluded from the analysis. Therefore, interactions from 17 trios with 51 students in classes with the inclusive program and 14 trios with 42 students in control classes were analyzed, including those present at both the pre-test and post-test.

### 2.2 Design

We have conducted a pre-post test intervention in order to test the impact of the inclusive program by comparing 4 classes with the inclusive program to 4 control classes without the inclusive program. Although the study design was not preregistered, it received approval from the ethics committee of the host university and the heads of the teaching departments. This approval allowed the collection of data in the schools based on the study’s description prior to implementation. Written informed consent was obtained from the parents, their teachers, and their headmasters.

#### 2.2.1 Independent variable

The main independent variable is the introduction of the inclusive program as described in section 1.5. Teachers from the control and treatment classes were all trained to cooperative learning before the intervention.

#### 2.2.2 Dependent variables

##### 2.2.2.1 Status among peers

Status was measured using a sociometric instrument inspired by Cohen and Lotan (1997) at the beginning and end of the school year. In determining status among peers, local characteristics, such as academic status and popularity are significant factors (Lotan, 2022). In the study by Cohen and Lotan (1997), students were asked to indicate the names of those in their class who were considered the “best at math and science” for academic status and those who were considered their “best friends” for social status. A scoring system ranging from 1 to 5, based on the quintiles of the classroom distribution, was proposed. The scores for academic and social status were then combined to create a “co-status score.”

A pilot study conducted in our specific context revealed that the original measure was problematic. Students found it uncomfortable and strange to indicate who the “best students” or “best friends” were. In order to avoid a competitive framing of the question, we adapted the measure. Instead, we provided a list with the names of all students in the class and asked students to indicate (a) the students in the class with whom they liked to work in groups (either a lot or a little) for school status, and (b) the students with whom they liked to play during free time like recess, lunch break (either a lot or a little) for social status. These measures allowed us to calculate a weighted status score, assigning 2 points for the highest intensity (liking a lot) and 1 point for the lower intensity (liking a little), while students who were not chosen received 0 points. The correlation between the scores of academic status and social status was high, with *r* = 0.83 for the pre-test and *r* = 0.85 for the post-test, *p* = 0.001. Consequently, we calculated the co-status by summing the two weighted scores, *M_pre-test_* = 38.11, *Min _pre-test_* = 13.00 and *Max _pre-test_* = 64.00; *M_post-test_* = 39.83, *Min _post-test_* = 0.00 and *Max _post-test_* = 74.00.

##### 2.2.2.2 Index of status problems

To document the existence of problem status in the classroom, an adapted index proposed by Cohen and Lotan (1997) was used. This index calculated the correlation coefficient (*Pearson r*) between the co-status scores of individual students and their observed average rate of peer task-related talk during work at learning centers. Cohen and Lotan (1995, 1997) conducted observations of students during structured cooperative work. They utilized a single indicator, “task-related talk,” which encompassed discussions related to the task at hand, cooperation among students, and discussions about individual roles. Additionally, they examined the role of the facilitator, a traditional role introduced within the complex instruction method (Cohen, 1994). However, in our study, a non-structured group work approach was intentionally introduced to examine whether status problems could arise when students were free to organize themselves as they wished. This allowed for variations not only in the quantity but also in the quality of student participation, as proposed by Buchs et al. (2018). To explore different types of participation that reflect a potential continuum related to status expression, a coding scheme was adopted, categorizing students’ participation into four categories:

(a) Exclusion: The student’s contribution is disregarded, ignored, or rejected by the group.
(b) Discrete participation: The student observes and follows the actions of groupmates without actively engaging, or is prompted by a groupmate to contribute.
(c) Co-construction: The student actively participates in verbal discussions related to the task content, organization, or promotes an inclusive environment that encourages the involvement of all students.
(d) Leadership: The student exhibits behaviors that limit others from participating, questions or negotiates others’ contributions, rejects input from others, or invites others to speak, react, or behave.

This continuum, from exclusion to leadership, gives insight concerning the severity of problem status; the two extreme categories being more severe. The video recordings were divided into 10-s segments, and each student’s actions were coded for each segment. As some groups completed the activity more quickly, the first 60 segments of 10 s each were coded for all groups. Inter-rater agreement was assessed, and after achieving satisfactory agreement (97% average agreement over 220 segments), only one coder was retained. The coding process was blind to the condition, timing of the video (pre-test or post-test), and student status information.

## 3 Results

### 3.1 Results regarding status among peers

The inclusive program was expected to enhance the status of students, with greater improvements in classes with the program compared to classes without the program (Hypothesis H1a). Additionally, it was hypothesized that the inclusive program would have a particularly positive impact on students’ status with low initial status (Hypothesis H1b). Table 1 indicates the evolution of students’ status among peer in the two conditions.

**Table 1:** Students’ status at the beginning and the end of the year with and without the inclusive program.

	With inclusive program		Without inclusive program	
	Pre-test Beginning of the year	Post-test End of the year	Pre-test Beginning of the year	Post-test End of the year
*M*	36.04	43.21	40.66	35.61
*SD*	8.96	8.47	11.76	13.89

M, Mean; SD, Standard deviation.

**Figure 1:**
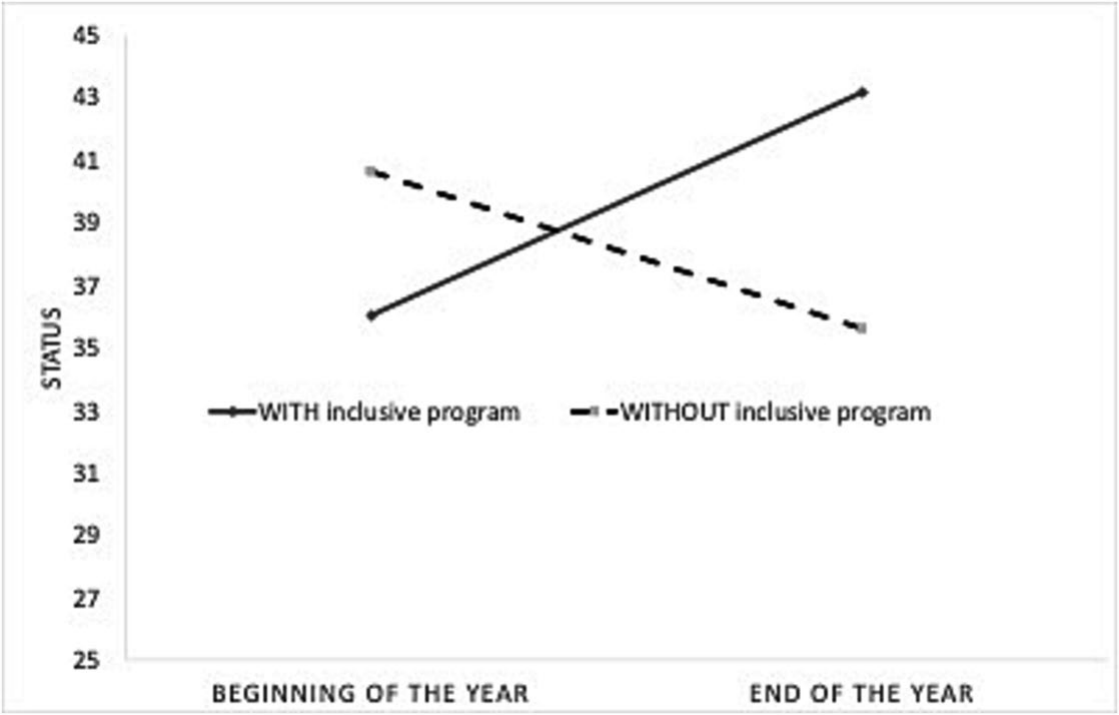
Evolution of students’ status from pre-test to post-test regarding the intervention (with and without inclusive program).

The repeated measures ANOVA analysis (Intervention X Time) revealed that the inclusive program did moderate the evolution of student status, as indicated by a significant interaction, *F*(1, 137) = 81.76, *p* < 0.001, *η^2^_p_* = 0.37. At the pre-test stage, students from control classes without the program exhibited higher status, *M* = 40.66, compared to students from targeted classes, *M* = 36.04, *p* < 0.01. However, by the end of the year, the situation reversed, with the inclusive program, students showed higher status, *M* = 43.21, than those from control classes, *M* = 35.61, *p* < 0.001. Furthermore, the inclusive program contributed to the improvement of students’ status from pre-test to post-test, *ΔM* = 7.17, *p* < 0.001, whereas status decreased in classes without the program, *ΔM* = −5.05, *p* < 0.001, see Figure 1.

Additionally, it was hypothesized that the inclusive program would have a particularly positive impact on students’ status who initially had low status (Hypothesis H1b). This hypothesis suggests a stronger negative relationship between initial status and the evolution of status with the inclusive program.

For H1b, a regression model was employed to examine the relationship between the evolution of status (dependent variable) and the initial status (centered), the intervention (coded as −1 for without intervention and + 1 for with intervention), and the interaction between the two as predictors. The results indicated that the effect of the inclusive program was significant, *b* = 5.60 *t* = 9.00, *p* < 0.001, as was the effect of initial status, *b* = −0.25, *t* = −4.16, *p* < 0.001. Crucially, the interaction between the intervention and initial status was found to be significant, *b* = −0.28, *t* = −4.73, *p* < 0.001. The model accounted for 50% of the variation in the evolution of status among peers.

**Figure 2:**
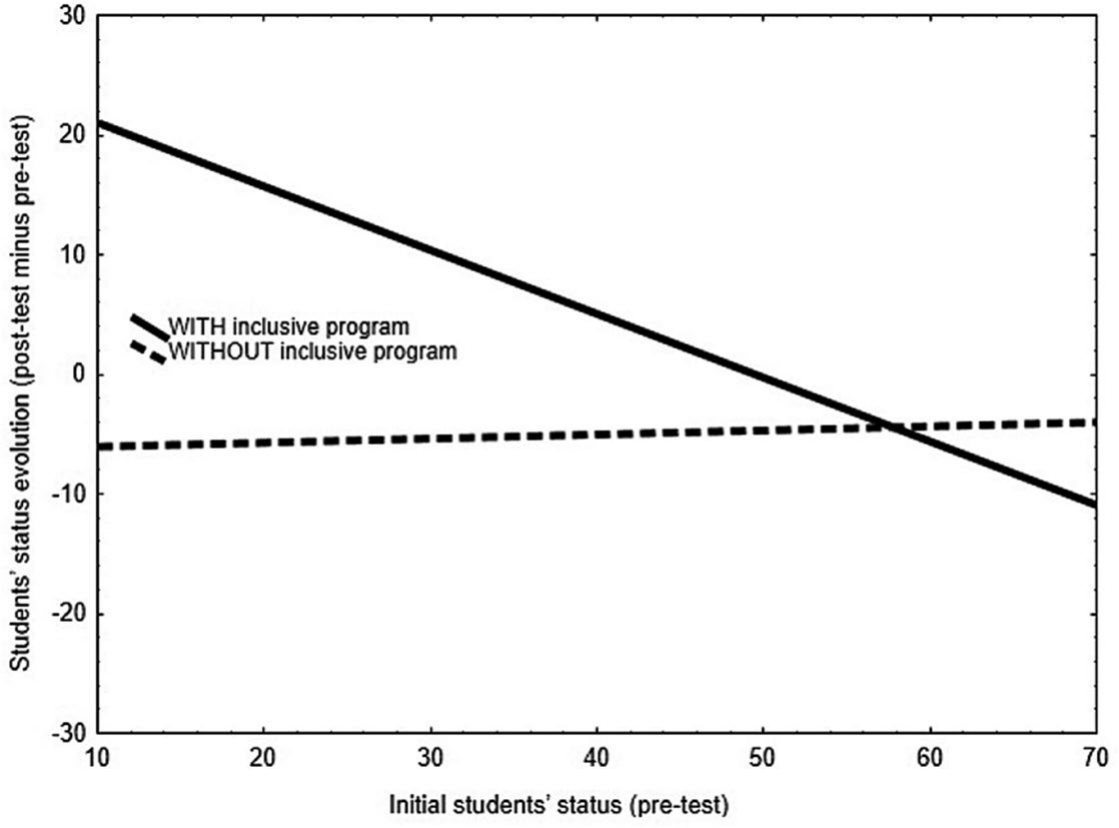
Students’ status evolution in fonction of the initial students’ status regarding the intervention (with and without inclusive program).

Figure 2 illustrates the interaction effect. In classes with the inclusive program, there was a significant negative association between the evolution of status and initial students’ status, *b* = −0.53, *t* = −5.83, *p* < 0.01, indicating a noteworthy positive evolution of status for students with low initial status. In contrast, without the inclusive program, the relationship between status evolution and initial status was not significant, *b* = 0.03, *t* = 0.44, ns. This suggests that in the absence of the program, students have maintained their status whether initially high or low.

For students with high initial status (+1SD), the effect of the intervention on their status evolution was less pronounced, *b* = 2.63, *t* = 2.95, *p* < 0.001 compared to students with average status, *b* = 5.60, *t* = 9.00, *p* < 0.001 or low initial status (−1SD), *b* = 8.59, *t* = 9.74, *p* < 0.001. This finding suggests that the inclusive program specifically benefits students with low initial status in terms of improving their status over time. It is important to note that, with the inclusive program, the status evolution turned negative for students who had a score higher than 50 in their initial status, i.e., for 4 out of 51 students.

### 3.2 Results regarding status-problems

The second series of hypotheses aimed to investigate the role of the inclusive program in the evolution of status problems. Status problems could be identified through: (a) positive relationships between status and assertive types of participation (e.g., high-status students being more likely to endorse leadership and engage in co-construction), and (b) negative relationships between students’ status and passive types of participation (e.g., low-status students being more likely to be discrete or excluded). Correlations between students’ status and participation are presented in Table 2. Due to the non-normality of the data regarding the types of participation (i.e., Being excluded, Discrete participation, and Leadership), correlations are reported for both the original data and the transformed data.

**Table 2:** Correlations (original and transformed data when required) between students’ status among peers and type of participation.

		All classes (*N* = 93)	Without inclusive program (*N* = 42)		With inclusive program (*N* = 51)	
		H2a	H2b	H2c	H2b	H2c
		Status (PRE-test) - Participation (PRE-test)	Status (POST-test) - Participation (POST-test)	Status (PRE-test) - Participation (POST-test)	Status (POST-test) - Participation (POST-test)	Status (PRE-test) - Participation (POST-test)
Being excluded	Original data	−0.26*	−0.32*	−0.39*	0.03	−0.01
	Transformed data^1^	−0.25*	−0.23	−0.26†	−0.03	−0.02
Discrete participation	Original data	−0.29**	−0.28†	−0.32*	0.19	−0.04
	Transformed data^1^	−0.15	−0.17	−0.29†	0.03	−0.08
Co-construction	Original data	0.14	0.10	−0.02	0.07	0.03
Leadership	Original data	−0.05	0.13	0.32*	−0.06	−0.14
	Transformed data^1^	0.02	0.25	0.35*	0.01	−0.08

^1^Transformed data (log) in order to respect condition for computing correlations (normality). **p < 0.01; *p < 0.05; ^†^*p* < 0.10.

*H2a*: At the outset of the academic year (pre-test), students in highly diverse classrooms may exhibit a pattern of status problems. To investigate this hypothesis, the relationship between students’ initial status (pre-test) and their type of participation at the beginning of the year (pre-test) was examined.

At the beginning of the academic year, the correlations observed across all classes suggested the presence of status problems, as indicated by negative correlations between initial status and more passive forms of participation. Students with lower initial status were more likely to experience exclusion, *r_original_* = −0.26, *p* = 0.01 and *r_transformed_* = −0.25, *p* = 0.01. Negative correlations were also found for discrete participation in the original data, *r_original_* = −0.29, *p* = 0.005, but these correlations were not significant with the transformed data, *r_transformed_* = −0.15, *p* = 0.14. Initial status showed no significant relationship with co-construction, *r_original_* = 0.14, *p* = 0.17 or leadership, *r_original_* = −0.05, *p* = 0.64, *r_transformed_* = 0.02, *p* = 0.88. This initial pattern was consistent with a dynamic of exclusion experienced by students with lower status among their peers.

*H2b*: By the end of the year, it is hypothesized that if status problems were present during the pre-test, they may persist in classrooms without the inclusive program, but would be diminished in classrooms with the inclusive program. Consequently, participation should not be associated with any specific status with the inclusive program. To test this hypothesis, the relationship between students’ final status (post-test) and their type of participation at the end of the year (post-test) was examined in the two conditions.

In the post-test phase, the students’ status at the end of the year was not correlated with the type of participation in the classes with the inclusive program. The correlations observed with the original data ranged from −0.09 > *r_original_* < 0.10, *p* > 0.48, and with the transformed data, the correlations ranged from −0.03 > *r_transformed_* < 0.19, *p* > 0.71. These findings illustrate an equal-status participation in the classes with the inclusive program.

In contrast, in the control classes, the correlations between status and participation were higher. The correlations with the original data ranged from −0.32 > *r_original_* < 0.10, while the correlations with the transformed data ranged from −0.23 > *r_transformed_* < 0.25. At the end of the year in the control classes, the pattern observed is consistent with the expectations in the case of status problems. There were negative correlations between status and passive participation, indicating that lower-status students were more likely to be excluded and adopt discrete participation. There was a positive correlation between status and assertive participation, indicating that higher-status students were more likely to participate in co-construction and assume leadership roles. However, it is worth noting that correlations with the transformed data were not significant.

*H2c*: At the end of the year, the inclusive program is expected to disrupt the connection between initial status (pre-test) and participation at the end of the year (post-test). In other words, the inclusive program should facilitate equal-status participation across different status levels, and initial status should no longer be linked to distinct types of participation with the inclusive program.

The final hypothesis examines whether students retained any trace of their initial status from their initial status (pre-test) when working with their classmates at the end of the year. The pattern of correlations appears consistent with persistent marker for lower-status students in the control classes. Negative correlations persisted between initial status and passive participation. Lower-status students were more likely to remain excluded at the end of the year, *r_original_* = −0.39, *p* = 0.01; *r_transformed_* = −0.26, *p* = 0.10, and engage in more discrete participation, *r_original_* = −0.32, *p* = 0.04; *r_transformed_* = −0.29, *p* = 0.06. However, when using transformed data, these correlations were not significant. Additionally, in the control classes, students with higher initial status continued to demonstrate more leadership at the end of the year, *r_original_* = 0.32, *p* = 0.04; *r_transformed_* = 0.35, *p* = 0.02. There was no relationship found between initial status and co-construction in either classes without the program, *r_original_* = −0.02, or the classes with the inclusive program, *r_original_* = 0.03.

In the classes with the inclusive program, no significant relationship was found between initial status and any form of participation, and the correlation coefficients were very weak, ranging from −0.13 > *r_original_* < 0.03 and from −0.02 > *r_transformed_* < −0.08 for transformed data. This suggests that students in these classes were more engaged in equal-status participation, regardless of their initial status.

## 4 Discussion

This study was framed within an inclusive education perspective that aims to facilitate a positive classroom experience for all students. This necessitates pedagogical approaches that foster quality relationships in the classroom and address existing barriers for certain students, ensuring their active participation. Aligned with the principles of intercultural education (Batelaan and van Hoof, 2006; Berry and Sam, 2013) and *Complex instruction* (Lotan, 2022), the inclusive program integrates the values of diversity, equality, and equitable participation (Buchs and Maradan, 2021).

Considering sociolinguistic diversity in classrooms, the objective of the program tested in this study was to provide students with equal opportunities to contribute, taking into consideration their competence in their family language. This program included activities that promote openness to others, openness to linguistic diversity, and multilingual cooperative activities. Multilingual cooperative activities were designed to necessitate the contribution of all students while acknowledging their specific linguistic skills. This one-year inclusive program was expected to (1) enhance students’ status among their peers, particularly for those who initially had low status, and (2) contribute to more equal-status participation in classroom activities.

The results demonstrated that this inclusive program moderated the evolution of students’ status. There was a significant increase in status with the implementation of the inclusive program, with students being more cited as play and work partners at the end of the year. This outcome may seem intuitive, considering that the students had spent a school year together in the same class. However, in classes where the inclusive program was not implemented, not only did status fail to improve, but they actually declined.

As predicted also, the inclusive program had a positive impact on students who initially had low status. It was specific to the inclusive program. In the absence of the program, students with low as well as high initial status experienced a similar stagnation in their status. The negative relationship found between initial status and changes in status with the inclusive program could lead to concerns about high-status students being penalized. However, results showed that the negative change in status for high-status students occurred only for a few students, those who had an initial status above 50 (4 students on 51). This result can be explained by the measurement method: since the students could name many classmates they wanted to play and work with, adding names may require removing others.

These results are a first important step regarding equity in highly diverse classrooms, considering that all teachers, from classes with and without the inclusive program, were previously trained in cooperative learning. Introducing the inclusive program based on multilingual cooperative activities that mobilize heritage or family languages is efficient for supporting the status of students at risk. These findings highlight the transformative potential of plurilingual cooperative activities, shifting from a deficit perspective where plurilingual students or those who do not speak French at home are viewed as lacking the necessary skills to fully participate in classroom life. Instead, these activities provide a platform for valuing and recognizing the skills of these students, both by their peers and teachers in lines with status treatment proposed by *Complex instruction* (Cohen, 1994; Lotan, 2022).

The second hypothesis proposed an additional step toward equity in highly diverse classrooms. It examined the status problems, specifically whether students’ status determined their participation. The initial pattern at the beginning of the year illustrated a status problem, with low-status pupils more likely to participate passively. Results suggested a dynamic of exclusion experienced by students with lower status. The correlations suggested that this pattern remained present at the end of the year without the inclusive program. The correlations in these classes were negative with passive participation types and positive with active participation types. This pattern was found for both pre-test status and post-test status. Caution is needed because the correlations were weak and non-significant for transformed data. Nevertheless, this pattern contrasted with the absence of correlation between status and type of participation at the post-test in classes where the inclusive program was implemented. Neither initial status nor status at the post-test were related to students’ participation in classes with the inclusive program, which evoked equal-status participation in these classes.

From a methodological aspect, this analyze of status problems is original and more precise than in previous studies. Previous research has examined average rates of peer task-related talk (Cohen and Lotan, 1995, 1997) and some types of participation (Buchs et al., 2018). In the present study, we refined the types of participation by examining qualitative types of participation that were supposed to have differentiated relationships with status. The pattern of results aligned with this proposition. In situations where status problems were expected, the correlations followed this pattern. At the beginning of the school year, students with low initial status participated passively, experiencing higher levels of exclusion and displaying more discreet behavior, highlighting potential initial status-related issues. This pattern persisted in control classes without the inclusive program, where low-status students were more likely to remain passive, while initially high-status students were more likely to become leaders. Even if they bring a new light on status problems, this methodology and associated patterns should be tested in future research to further validate their significance. One major challenge in our results was the non-normality of the data. Results indicated that with the required statistical transformations, the strength of correlations was reduced, rendering them non-significant.

Thus, this inclusive program and the guidelines on which it is based are promising perspectives to teach in diverse classrooms. Nevertheless, it remains demanding and time consuming, what constitutes a major barrier for teachers (Buchs et al., 2017; Abramczyk and Jurkowski, 2020). Therefore, future directions should address the question by providing efficient support for teachers for properly introduce cooperative structure more easily compatible with teacher daily constraints. Structural approach (Kagan, 2021) introduces some simple procedures in order to propose simultaneous interactions as well as equal participation. Easy enough for becoming cooperative routines in daily teaching, these structures have the potential to reinforce students’ in-class participation. Preliminary results underlined positive effects for students in general (Kothiyal et al., 2013; Reddy et al., 2015) as well for the participation of shy students (Mundelsee and Jurkowski, 2021). Additional research is needed to investigate the potential of this structural approach in order to sustain participation of students with low initial status, especially in highly diverse classrooms. This could be an opportunity to sustain the quantity of cooperative implementation while ensuring equal-status participation.

## 5 Conclusion

To rely on cooperative learning to support inclusion induces two paradoxes. Firstly, while cooperative learning is the most cited way to promote inclusion (Juvonen et al., 2019), there is a low implementation of cooperative learning in regular classrooms (Baines et al., 2003; Abrami et al., 2004; Pianta et al., 2007; Buchs et al., 2017). Secondly, there is the potential for counterproductive effects of cooperative learning if status issues are not actively addressed (Cohen, 1994; Lotan, 2022).

To address the first paradox, it is important to provide teachers with concrete strategies that introduce regular cooperative activities. Teachers claim their interest for lesson examples and teaching material (Abramczyk and Jurkowski, 2020). It is crucial to propose such examples, built in collaboration with teachers, and to empirically demonstrate their effectiveness in the context of diversity. Such results bring confidence to implement cooperative learning in classroom with large diversity and confidence is essential for teacher attitudes toward inclusive practices (Desombre et al., 2019; Abramczyk and Jurkowski, 2020; Jury et al., 2023) and inclusive schools. Moreover, the inclusive program adopted a collective approach of classroom management, avoiding labels or categorization of targeted students. This aligns with the implementation of a Universal Design for Learning (UDL) approach (Rose and Meyer, 2002; Katz, 2013), which is also often perceived as challenging or even impossible to implement.

To address the second paradox requires a high quality of implementation for sustaining equal participation (Abramczyk and Jurkowski, 2020). According to our results, it is essential to train teachers regarding the consequences of status problems and to empower them with tools able to create equal-status interactions that enhance all students’ learning experiences and outcomes (Lotan and Holthuis, 2021). Our study bring knowledge about the status problems and develop an effective tool to foster equal-status interaction in context of high diversity classroom.

These two paradoxes may be addressed by proposing dedicated material and program as well as targeted training for pre-service and in-service teachers. This was the objective of the inclusive program that has been developed and tested. The inclusive program we proposed aligns with the process of engineering suggested by Lotan and Holthuis (2021). This process includes preparing teachers, designing and constructing curricula, developing status interventions, and constantly checking and testing proposed responses against theoretical claims. The inclusive program was introduced to teachers as ready-to-use activities. The research team provided the activities with materials, scripts, and structuration. Teachers then implemented them in their own classrooms. While there were some challenges regarding the time required, there were no reported difficulties in implementing the program. Both students and teachers provided positive feedback. This is encouraging regarding the paradoxes discussed, cooperative learning is likely to support inclusion if carefully structured. By acknowledging specific status characteristics (linguistic skills) in multiple abilities tasks, the program reinforced a positive perception of students within the class (Lotan, 2022). To our knowledge, this program is the first to overcome status problems in classrooms with such large sociolinguistic diversity. Teachers are often skepticism and lack of confidence when they have to teach in such diverse classroom (Jury et al., 2023). This study shows that such program can outcome status problems through cooperative activities. Such status modification in classroom with large diversity is particularly important for the inclusive perspective (UNESCO, 2009; Forslund Frykedal and Hammar Chiriac, 2018; Farmer et al., 2019; UNESCO, 2019).

This program is also an outstanding method to promote positive intergroup contact between diverse students. The program meets the conditions for reducing prejudice (Pettigrew and Tropp, 2006). Indeed, it supports openness to diversity and carefully structures conditions for students from different groups to successfully cooperate toward a common goal. It ensures equal status between the groups with a normative climate of tolerance and empathy. Such conditions of intergroup contacts have the potential to improve stereotypes assigned to the groups these students represent (Hewstone et al., 2018) and even to other minority groups (Pettigrew, 1997). Such positive intergroup contact also increases willingness for intergroup contact outside the schools (Reimer et al., 2022). This can accelerate teaching language learning which is crucial for rapid inclusion of migrant students in mainstream classrooms. Therefore, addressing actively students’ status problems within diverse classrooms is not only a key factor for classroom social interactions, it is also a way to accelerate the inclusion of migrant students in schools, and to develop coexistence in our multicultural societies.

## Data availability statement

The raw data supporting the conclusions of this article will be made available by the authors, without undue reservation.

## Ethics statement

The studies involving humans were approved by Ethics committee from University of Geneva. The studies were conducted in accordance with the local legislation and institutional requirements. Written informed consent for participation in this study was provided by the participants’ legal guardians/next of kin.

## Author contributions

CB: Conceptualization, Formal analysis, Funding acquisition, Methodology, Supervision, Writing – original draft, Writing – review & editing. NM: Writing – review & editing. MH: Formal analysis, Writing – review & editing.

## Footnotes

^1^https://complexinstruction.stanford.edu/

^2^The Swiss Federal Statistical Office, https://www.bfs.admin.ch.

^3^Genevan office for research in education, https://www.ge.ch/organisation/service-recherche-education.
